# Right atrial and pulmonary cement embolization following vertebral laminectomy: An incidental finding

**DOI:** 10.1002/ccr3.7193

**Published:** 2023-04-16

**Authors:** Solmaz Borjian, Mohammad Amin Borjian, Aryan Ayati, Arezou Zoroufian

**Affiliations:** ^1^ Tehran Heart Center, Cardiovascular Diseases Research Institute Tehran University of Medical Sciences Tehran Iran; ^2^ Department of Advanced Echocardiography Tehran Heart Center, Tehran University of Medical Sciences Tehran Iran; ^3^ School of Medicine Iran University of Medical Sciences Tehran Iran

**Keywords:** cement, complication, echocardiography, embolization, vertebroplasty

## Abstract

Right heart cement embolization is a rare but potentially life‐threatening complication of vertebroplasty surgeries. Transthoracic echocardiography is the first‐line imaging modality for detecting cement particles in cardiac chambers. Anticoagulation treatments or surgical interventions are necessary, depending on the patient's condition.

## Graphical abstract

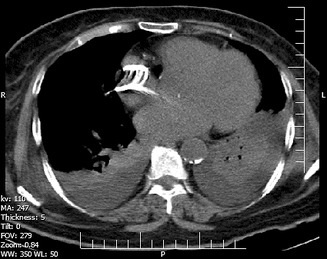

## 1 INTRODUCTION

Right heart cement embolization is a rare complication of vertebroplasty surgeries. It can cause life‐threatening conditions such as ventricular rupture if left undetected. In this case report, we present a 57‐year‐old woman with a history of laminectomy diagnosed with right atrial cement embolization during echocardiography as an incidental finding.

Cement embolization is the main complication of vertebroplasty due to the embolization of poly methyl methacrylate (PMMA) in the paravertebral venous circulation, followed by the pulmonary vasculature or the right heart.^1^ PMMA embolization can lead to cerebral embolization, pulmonary embolization, right heart embolization, and renal artery embolization.^1^ Pulmonary cement embolism is more common than right heart embolization and could be more lethal and life‐threatening. Right heart embolization could result in ventricular perforation and tamponade or damage to the tricuspid valve in milder cases.^2^

The reported frequency rate of pulmonary cement embolization following vertebroplasty ranges between 4.6% and 26.9%,^1^ and right heart embolization seems much rarer (≈3.9%).^3^ Some cases of right heart cement embolization are asymptomatic or have nonspecific symptoms diagnosed as an incidental finding in imaging studies for other reasons; hence, they are subjected to conservative treatment and follow‐up.^3^

To prevent thrombotic events, oral anticoagulation therapy is usually recommended in asymptomatic patients with small fragments of cement (pulmonary or right heart) embolization.^4^ In symptomatic patients at risk of ventricular perforation, the surgical extraction of cement as a foreign body is the gold‐standard treatment strategy.^4^

Cardiac perforation and tamponade are catastrophic manifestations of this complication, necessitating urgent surgery.^5^

This case report presents a patient with right atrial cement embolization originating from a recent laminectomy surgery. The embolization was diagnosed as an incidental finding during angiography and the subsequent echocardiography.

## 2 CASE DESCRIPTION

A 57‐year‐old woman with a history of typical chest pain associated with elevated cardiac troponin was referred to our tertiary cardiac center with a diagnosis of non‐ST‐elevation myocardial infarction (NSTEMI) for further evaluation and coronary angiography.

In her past medical history, the patient had a history of breast cancer, treated completely many years earlier, and a history of vertebral column laminectomy (lumbar) 2 weeks before her new cardiac symptoms and hospital admission.

Limited bedside transthoracic echocardiography was performed with a focus on the left and right ventricular systolic functions and regional wall motion abnormalities.

**FIGURE 1:**
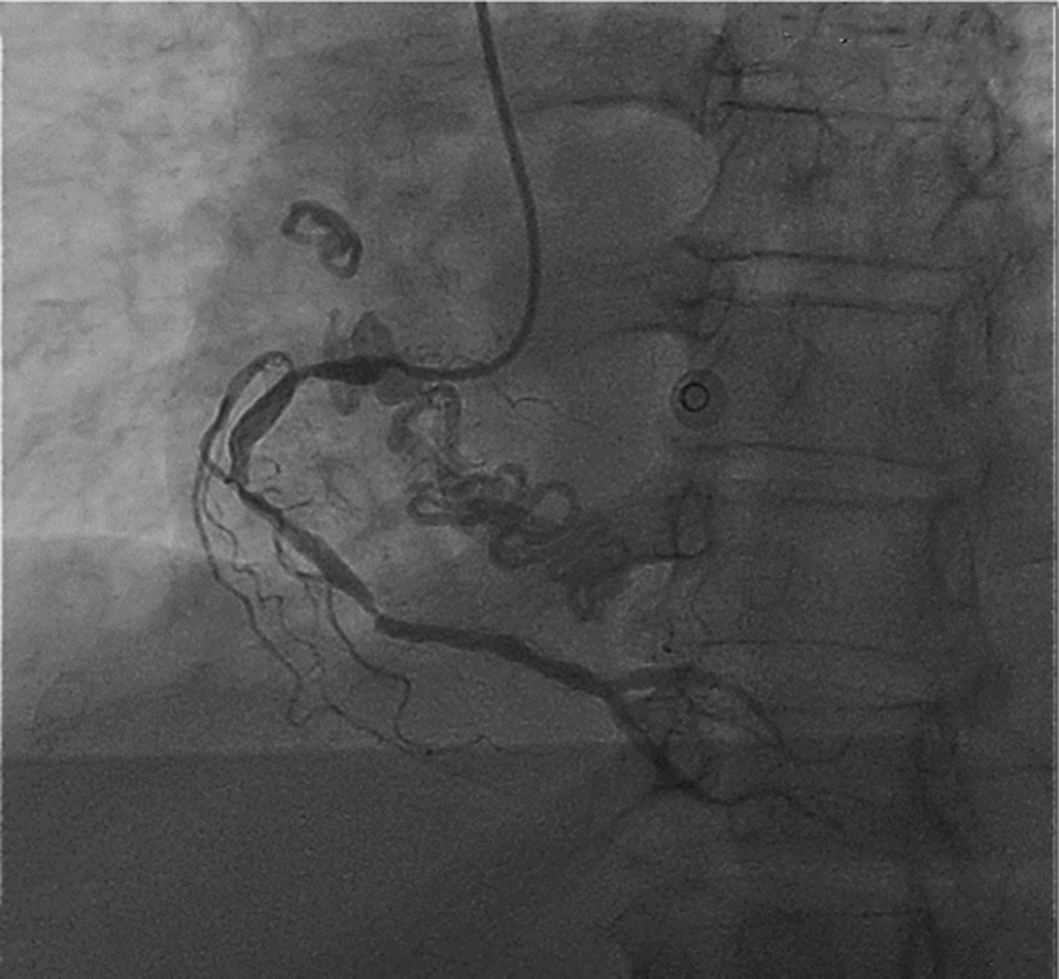
The right coronary artery window shows a spiral‐shaped density in the right heart and the pulmonary vascular bed.

Coronary angiography, performed as an early invasive approach, showed triple‐vessel coronary artery disease. During the angiography and fluoroscopy, two large mobile spiral calcified masses were noticed in the right heart and a smaller one in the right lung moving with cardiac and respiratory motions (Figure 1).

**FIGURE 2:**
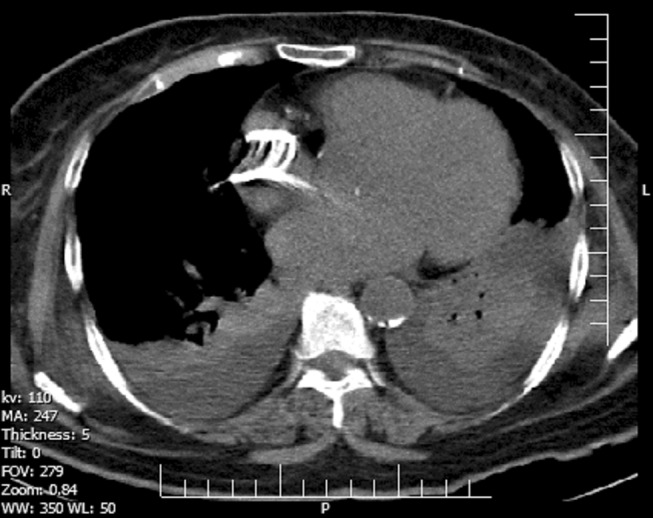
The mediastinal window in the spiral chest computed tomography scan shows a high‐density focal point with a streaky artifact in the right atrium, consistent with cement embolization.

**FIGURE 3:**
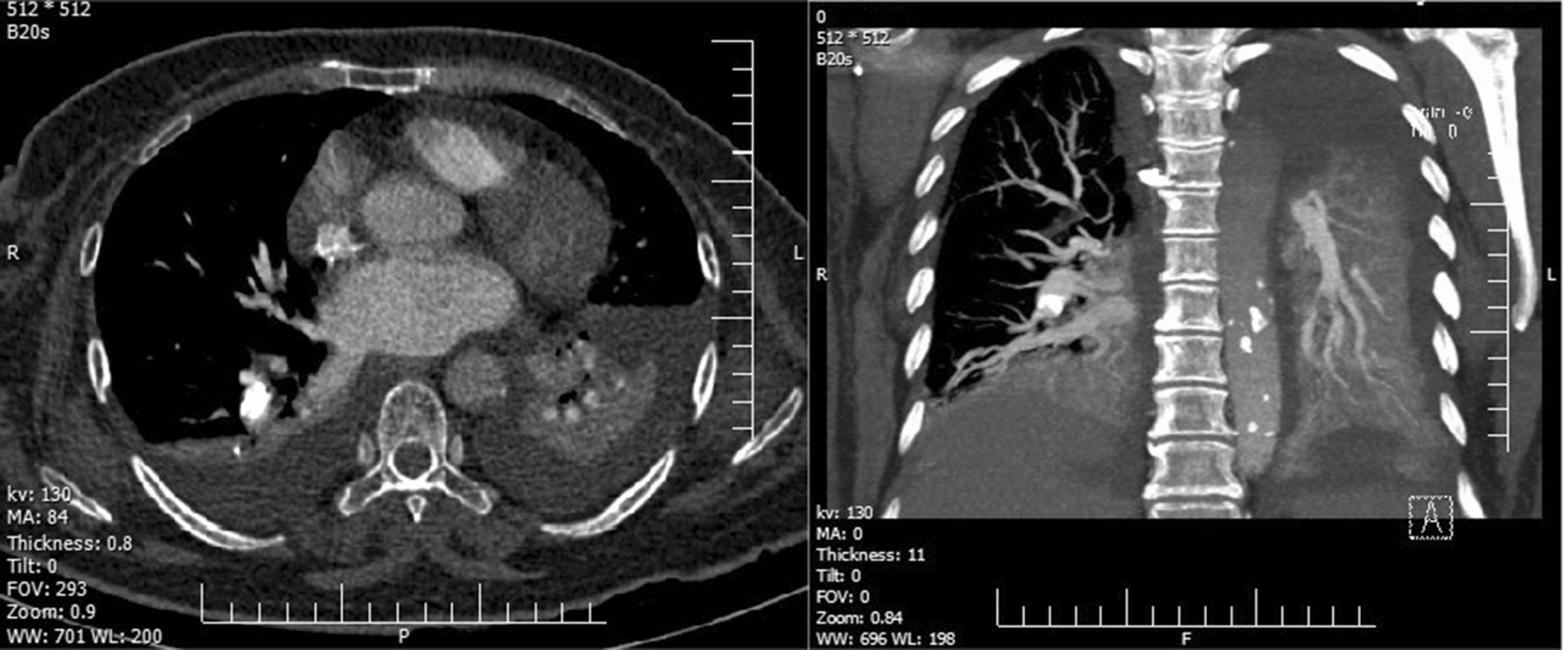
The spiral chest computed tomography angiography (a: axial; b: coronal maximum intensity projection) shows a hyperdense filling defect in the posterior basal branch of the right pulmonary artery, consistent with cement embolization.

Further evaluation by spiral chest computed tomography (CT) scan and pulmonary CT angiography revealed large foreign bodies in the lung and the heart (Figures 2 and 3).

**FIGURE 4:**
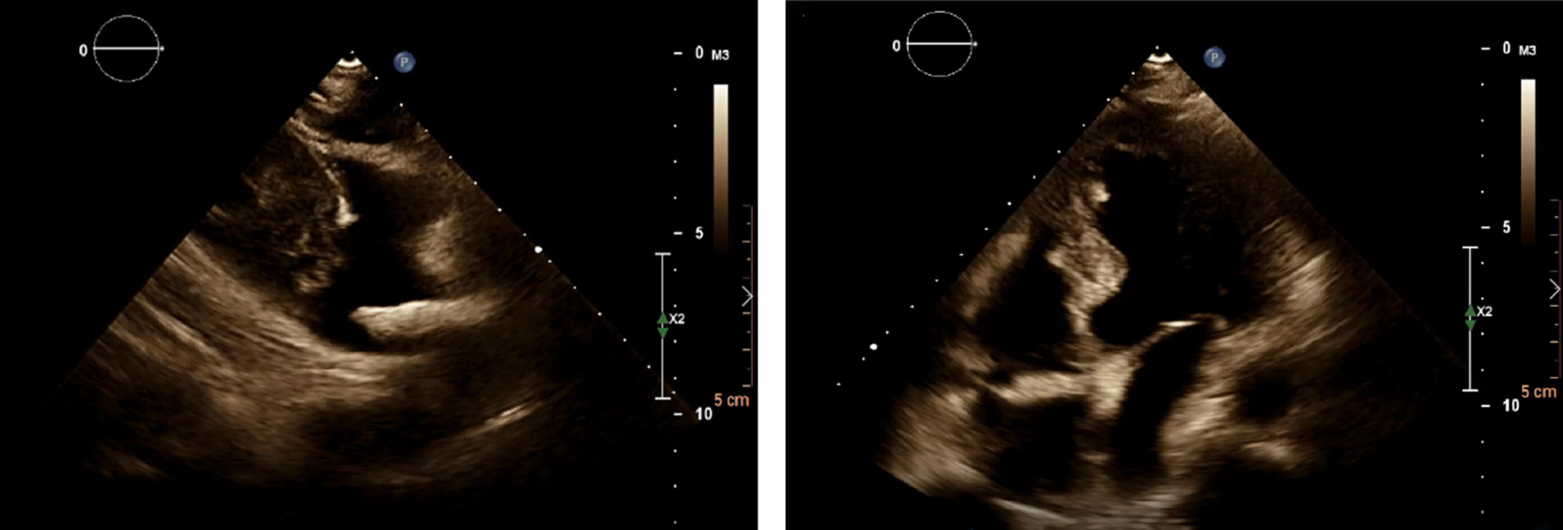
The transthoracic echocardiography of the right ventricular inflow in the four‐chamber view shows an elongated irregular hypermobile echodense mass in the right atrium.

**FIGURE 5:**
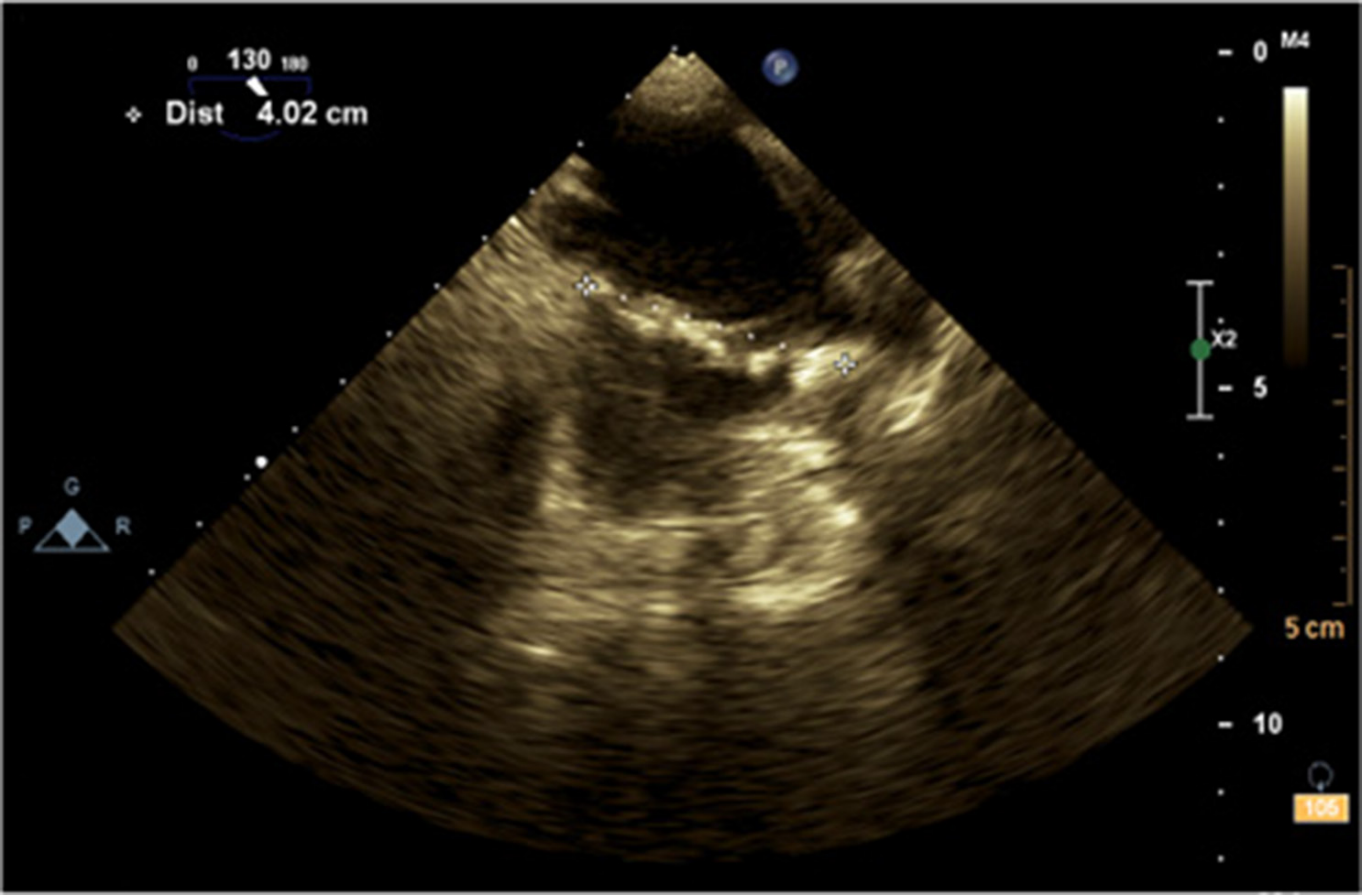
The transesophageal echocardiography in the midesophageal 130° view shows an elongated (4 cm in length) irregular hypermobile echodense mass in the right atrium.

**FIGURE 6:**
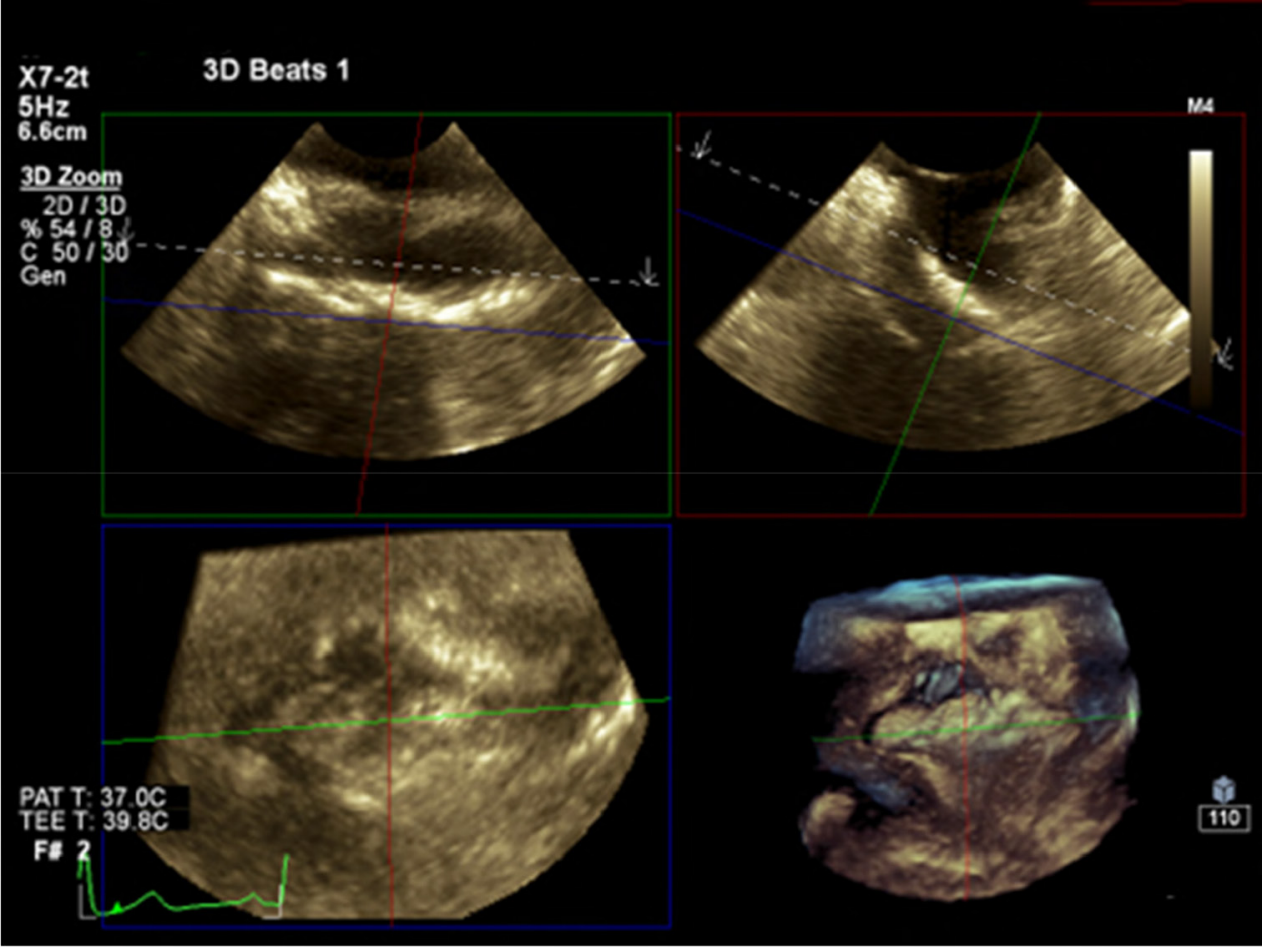
The 3D transesophageal echocardiography shows a multiplanar reconstruction of the elongated irregular hypermobile echodense mass in the right atrium.

**FIGURE 7:**
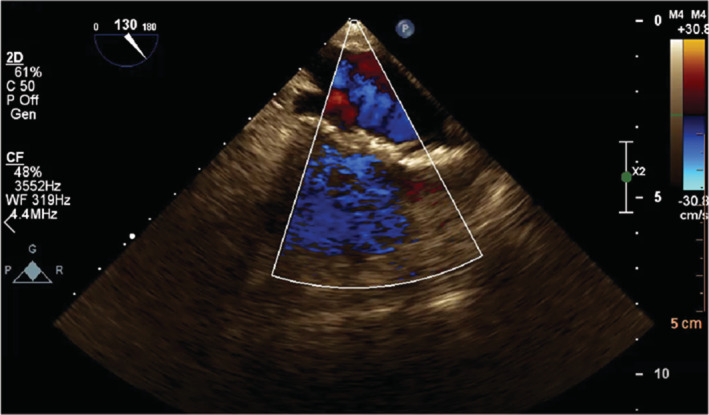
The transesophageal echocardiography in the midesophageal 130° (color mode) of the right atrium shows an elongated irregular hypermobile echodense mass.

**FIGURE 8:**
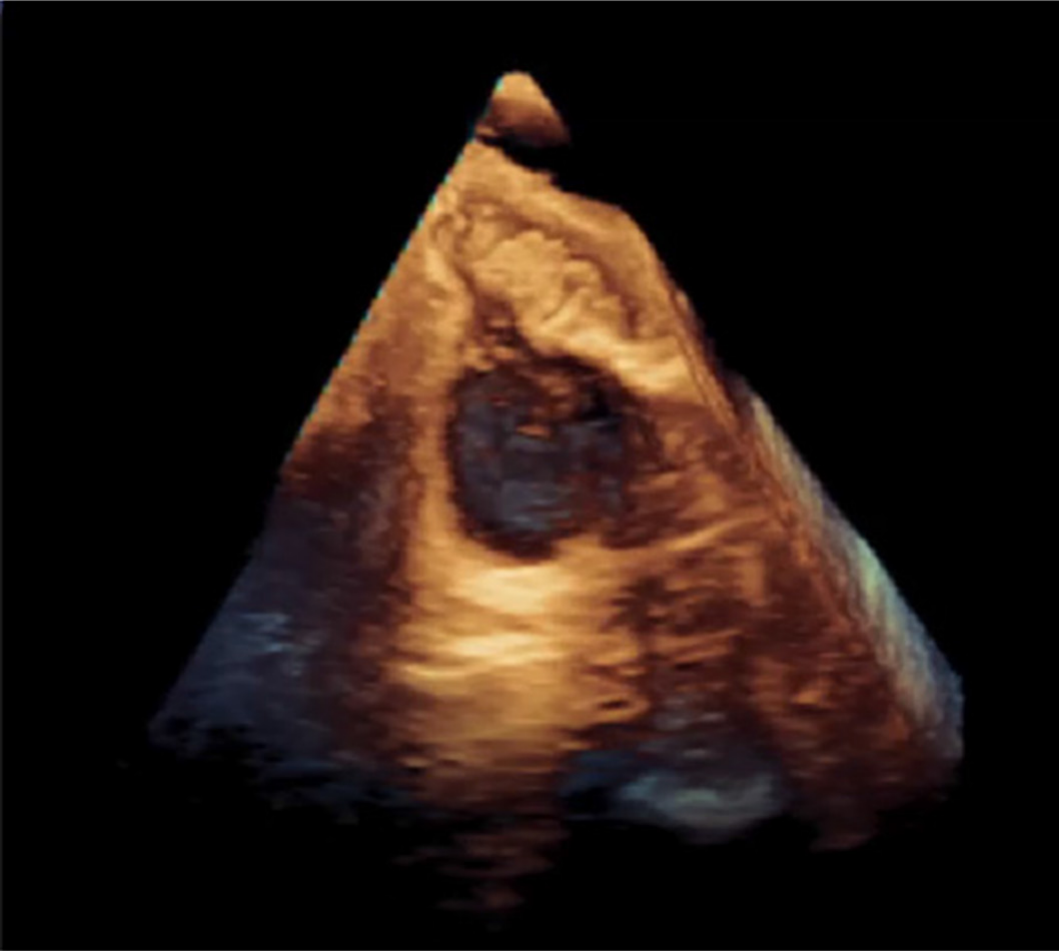
(Video S1): The 3D transesophageal echocardiography in the midesophageal 110° (live 3D image) shows the elongated irregular hypermobile spiral‐shaped mass in the right atrium.

We performed transthoracic and 3D transesophageal echocardiography (Figures 4, 5, 6, 7, 8) and found a large calcified mass inside the right atrial appendage and the right atrium (Video S1).

Given the patient's history of chemotherapy, we considered the probability of the presence of the remnant of a chemotherapy superior vena cava port with organized thrombus formation around it. Nevertheless, her family ruled out this likelihood stating that the patient had never undergone portal implantation. Subsequently, on account of her recent laminectomy, we considered the less likely diagnosis of probable cement embolization in the right atrium and the pulmonary vasculature (distal peripheral arteries).

We scheduled the patient for open‐heart surgery to remove the foreign body (cement) from the right atrium. However, the patient and her family refused consent for open‐heart surgery. Thus, the patient was discharged with anticoagulation therapy and was advised to return for 1‐month follow‐up echocardiography to evaluate the emboli development.

The patient failed to return for follow‐up visits. Further follow‐up contacts indicated that the patient was hospitalized for septicemia originating from a surgical site infection and expired due to organ failure caused by the septicemia 3 months after the visit to our center.

## 3 DISCUSSION

Cement embolization with PMMA via the paravertebral venous system following vertebroplasty is the most significant complication of this noncardiac surgery.^1^ The common embolization site is the pulmonary vasculature with small cement fragments. Although, rare embolization cases have been reported in other vasculature systems, including the brain, the heart, and the kidneys.^1^

The literature features a few case reports of cement embolization.^1^ In a case report by Lim et al.,^6^ intraarterial thrombus was reported as a late complication 5 years after percutaneous vertebroplasty. Wang et al.^3^ also reported a right‐heart cement embolization case 15 months after percutaneous vertebroplasty. Several recent cases were diagnosed with cement embolization after ventricular perforation,^4^, ^5^ causing mortality in one of the cases reported by Diab et al.^2^

Imaging modalities such as chest X‐rays and CT scans are the first steps toward an accurate diagnosis.^3^ Pulmonary CT angiography is the gold‐standard diagnostic modality for pulmonary vasculature.^3^ Two‐dimensional transthoracic echocardiography can be employed as the first‐line diagnostic approach to evaluate cardiac chambers and intracardiac embolization. Three‐dimensional echocardiography and transesophageal echocardiography could be the best diagnostic instruments to detect cardiac cement embolization inside the cardiac chambers.^3^

The contrast agents used in CT angiography might hide cement particles in the pulmonary vascular bed. Thus, simple chest X‐rays and spiral chest CT scans (without contrast injection) are essential in patients with a history of vertebroplasty to investigate possible cement embolization. Transthoracic echocardiography constitutes the first‐line imaging study for detecting cement particles in cardiac chambers and complications.

Most hitherto‐reported cases are asymptomatic or mildly atypically symptomatic cases in which mass detection was incidental. This group of patients could receive conservative treatment with oral anticoagulation drugs; nevertheless, in other symptomatic cases with such life‐threatening signs as ventricular perforation and tamponade, invasive surgical approaches, and cement extraction are deemed the gold‐standard treatment.^7^

Our cement embolization case was diagnosed incidentally, as well, because the patient was referred for coronary evaluation in an NSTEMI setting. Given her triple‐vessel disease, we recommended open‐heart surgery; otherwise, we could have chosen conservative oral anticoagulation therapy alone.

## 4 CONCLUSION

If left untreated, right heart cement embolization is a rare but potentially life‐threatening complication of vertebroplasty surgeries. This condition remains asymptomatic and can be detected as an incidental finding. Transthoracic echocardiography constitutes the first‐line imaging modality for detecting cement particles in cardiac chambers and their complications. Anticoagulation treatments or surgical interventions are necessary, depending on the patient's condition.

## AUTHOR CONTRIBUTIONS

**Solmaz Borjian:** Conceptualization; writing – original draft. **Mohammad Amin Borjian:** Data curation; visualization. **Aryan Ayati:** Writing – review and editing. **Arezou Zoroufian:** Conceptualization; supervision; writing – review and editing.

## FUNDING INFORMATION

None.

## CONFLICT OF INTEREST STATEMENT

All authors declare that there were no competing interests.

## CONSENT FOR PUBLICATION

All authors have consented for publication of the article.

## CONSENT

Written informed consent was obtained from the patient to publish this report in accordance with the journal's patient consent policy.

## Supporting information


Video S1.

## Data Availability

All data associated with the article is available if required.
